# Abundance and physiology of dominant soft corals linked to water quality in Jakarta Bay, Indonesia

**DOI:** 10.7717/peerj.2625

**Published:** 2016-11-29

**Authors:** Gunilla Baum, Indra Januar, Sebastian C.A. Ferse, Christian Wild, Andreas Kunzmann

**Affiliations:** 1 Department of Ecology, Leibniz Center for Tropical Marine Ecology, Bremen, Germany; 2 Faculty of Biology and Chemistry, University of Bremen, Bremen, Germany; 3 Indonesian Research Center for Marine and Fisheries Products Processing and Biotechnology, Jakarta, Jakarta Pusat, Indonesia

**Keywords:** Pollution, Coral reefs, Phase shifts, Eutrophication, Megacity, Photosynthetic yield, Electron transport system (ETS) activity, *Nephthea* spp., *Sarcophyton* spp.

## Abstract

Declining water quality is one of the main reasons of coral reef degradation in the Thousand Islands off the megacity Jakarta, Indonesia. Shifts in benthic community composition to higher soft coral abundances have been reported for many degraded reefs throughout the Indo-Pacific. However, it is not clear to what extent soft coral abundance and physiology are influenced by water quality. In this study, live benthic cover and water quality (i.e. dissolved inorganic nutrients (DIN), turbidity (NTU), and sedimentation) were assessed at three sites (< 20 km north of Jakarta) in Jakarta Bay (JB) and five sites along the outer Thousand Islands (20–60 km north of Jakarta). This was supplemented by measurements of photosynthetic yield and, for the first time, respiratory electron transport system (ETS) activity of two dominant soft coral genera, *Sarcophyton* spp. and *Nephthea* spp. Findings revealed highly eutrophic water conditions in JB compared to the outer Thousand Islands, with 44% higher DIN load (7.65 μM/L), 67% higher NTU (1.49 NTU) and 47% higher sedimentation rate (30.4 g m^−2^ d^−1^). Soft corals were the dominant type of coral cover within the bay (2.4% hard and 12.8% soft coral cover) compared to the outer Thousand Islands (28.3% hard and 6.9% soft coral cover). Soft coral abundances, photosynthetic yield, and ETS activity were highly correlated with key water quality parameters, particularly DIN and sedimentation rates. The findings suggest water quality controls the relative abundance and physiology of dominant soft corals in JB and may thus contribute to phase shifts from hard to soft coral dominance, highlighting the need to better manage water quality in order to prevent or reverse phase shifts.

## Introduction

Coral reefs worldwide are characterized by a considerable loss in coral cover and species diversity (Bellwood et al., 2004; Bruno & Selig, 2007). The degradation of coral reefs is often related to declining water quality linked to eutrophication and pollution as a result of urban run-off, which carries large amounts of domestic wastes and industrial effluents (Fabricius, 2005; van Dam et al., 2011). Eutrophication has been proposed as the main stress factor for many reefs worldwide (GESAMP, 2001). For example, long-term monitoring data from the Great Barrier Reef show that the overall reduction in total coral cover by 70% is mainly due to eutrophication (Bell, Elmetri & Lapointe, 2014).

A growing body of literature suggests that the degradation of coral reefs is often associated with shifts in the benthic community to new compositions (e.g. Done, 1982; Hughes, 1994). Phase shifts on coral reefs are usually associated with shifts from hard coral-dominated to macroalgae-dominated communities (Nyström, Folke & Moberg, 2000; Szmant, 2002; Hughes et al., 2007). However, shifts to reefs dominated by other benthic organisms such as sponges, corallimorpharians, zoantharians and soft corals have been reported as well (Chou & Yamazato, 1990; Fox et al., 2003; Ward-Paige et al., 2005). To date, these shifts have received less attention, and the underlying mechanisms are still poorly understood (Norström et al., 2009). Soft corals (Octocorallia) represent a diverse and widespread benthic group within coral reefs in the Indo-Pacific (Dinesen, 1983; Benayahu, 1997; Benayahu et al., 2004) and are important for reef structure and function (Cary, 1931). Studies on coral–macroalgae shifts suggest that those shifts are caused by loss of top-down control as a result of overfishing (Hughes et al., 2007; Rasher et al., 2012). In contrast, phase shifts to sponges, corallimorpharians and soft corals may be driven by bottom-up control and soft corals may be driven by bottom-up control and reduction in water quality (Holmes et al., 2000; Norström et al., 2009). However, the literature is unclear whether soft corals are more tolerant towards declining water quality compared to hard corals (Dinesen, 1983; Fabricius & De’ath, 2004). For instance, Fabricius & De’ath (2004) found that soft coral species richness declined up to 60% along a gradient of increasing NTU, while other studies found a higher tolerance of soft corals towards high sedimentation rates (McClanahan & Obura, 1997). In addition, there is considerably more knowledge available on hard-coral physiology than for soft corals, for instance on how the metabolism of soft corals is influenced by anthropogenic stress and whether soft corals react differently than hard corals on a physiological level. Such knowledge is however crucial to understand the conditions, such as for example reduced water quality, and underlying mechanisms that drive phase shifts to soft coral dominance, and is needed to improve management strategies for coral reefs (Folke et al., 2004).

Two promising indicators for metabolic stress responses in marine organisms to declining water quality are the photosynthetic capacity and electron transport system (ETS) activity (Jones, Kildea & Hoegh-guldberg, 1999; Fanslow, Nalepa & Johengen, 2001; Lesser, 2013; Maes et al., 2013). Photosynthetic capacity can be determined though the quantum yield of linear electron transport (i.e. photosynthetic yield = delta F/Fm’). ETS activity has been mainly used as an indicator for metabolic condition in zooplankton (Båmstedt, 1980; Gómez, Torres & Hernández-León, 1996) and fishes (Ikeda, 1989; Lannig et al., 2003), but only few studies have used it for marine invertebrate species such as mussels (Fanslow, Nalepa & Johengen, 2001; Nahrgang et al., 2013), and to our knowledge no studies have measured ETS in corals. The ETS is a multi-enzyme complex in the respiratory chain in the mitochondria during which electrons are passed along numerous enzymes and energy is generated for oxidative phosphorylation and adenosine triphosphate (ATP) synthesis. The synthesis and degradation of these macro-enzymes depends on the respiratory requirements of the organism and therefore by measuring ETS activity, a time-averaged value of the maximum oxygen uptake rate potential is given. Since ETS activity adjusts to changes in environmental conditions over several days and weeks, short-term fluctuations and experimental factors are less influential than for direct measurements of respiration (Båmstedt, 1980; Cammen, Corwin & Christensen, 1990). Both ETS activity and photosynthetic yield can increase in organisms exposed to pollution to compensate for stress effects (i.e. produce more ATP) or decrease due to toxic effects (van Dam et al., 2011).

With around 25 million inhabitants (Brinkhoff, 2011), the Greater Jakarta Metropolitan Area is the 2nd largest urban agglomeration in the world (United Nations, 2014). Located in front of Jakarta Bay (JB), the Kepulauan Seribu (“Thousand Islands”) chain represents an ideal area to assess the effects of multiple stressors on coral reef organisms. Various human-induced marine and coastal environmental problems such as high sediment load, water pollution, depletion of fishery resources, seafood contamination, loss of habitat, coastal littering as well as eutrophication have caused severe degradation of coral reefs in JB and the Outer Thousand Islands. Localized effects of anthropogenic stressors appear to have led to a spatial patchwork of differentially degraded reefs (Rachello-Dolmen & Cleary, 2007; Baum et al., 2015). Although reefs within the bay once had thriving coral communities (Verstappen, 1953; Arifin, 2004; van der Meij, Suharsono & Hoeksema, 2010), they are now dominated by sand, rubble and algae, with a current hard coral cover of < 5% for nearshore reefs within JB. Mid- and offshore reefs along the Thousand Islands have highly variable reef conditions (< 20% hard coral cover to 50%) (Cleary et al., 2014; Baum et al., 2015). Considering that coral reefs are of huge economic and environmental importance in the area, supporting fisheries and tourist sectors and providing habitats with high productivity and diversity, there is a growing need to understand coral reef functioning.

In order to increase our understanding of shifts towards soft coral dominance in reefs exposed to multiple anthropogenic stressors, this study aimed to answer the following research questions: 1) How does distance to Jakarta influence key water quality parameters; 2) How does distance to Jakarta (i.e. declining water quality) influence live benthic cover in local coral reefs and do hard or soft corals dominate; 3) Does water quality affect photosynthesis and ETS activity of two dominant soft coral genera in the area, *Sarcophyton* spp. (Family: Alcyoniidae) and *Nephthea* spp. (Family: Nephtheidae)? Which water quality parameters affect the metabolic condition of these soft corals? We hypothesize that closer to Jakarta a) water quality is reduced b) soft coral dominance of the living benthos occurs more frequently and c) the photosynthesis and ETS activity in soft corals are negatively affected by reduced water quality. In order to answer these questions, a combination of benthic surveys, water quality assessments, and physiological measurements were carried out.

## Material and Methods

### Study area

**Figure 1:**
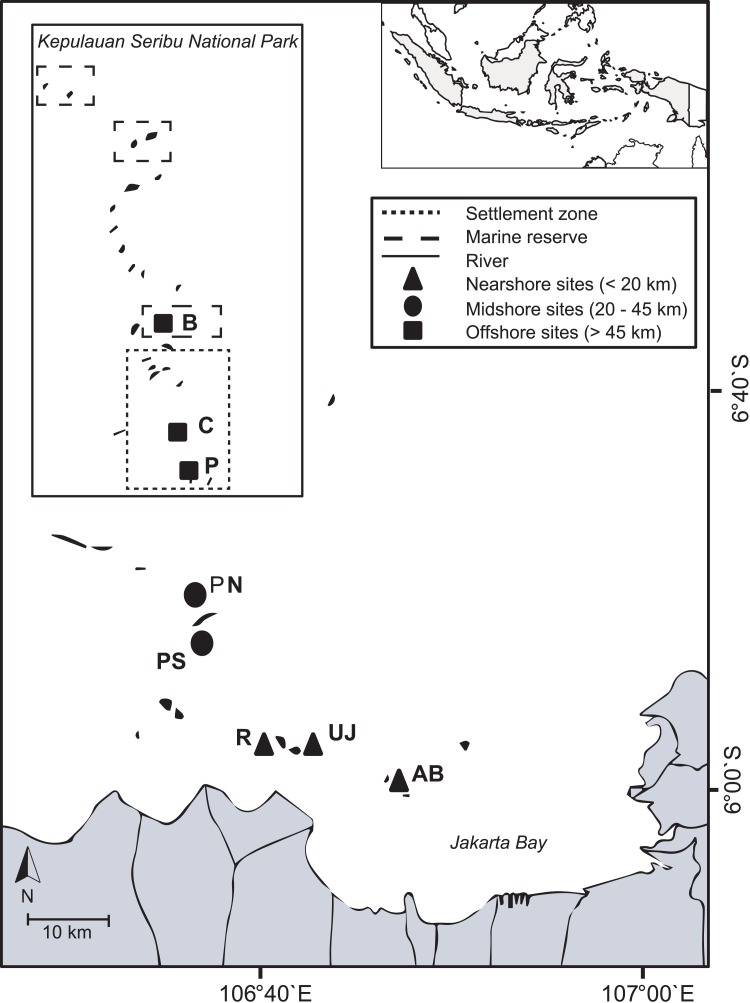
Study area. The map includes boundaries of the Thousand Islands Marine National Park and study sites from nearshore reefs (within Jakarta Bay), as well as from the outer Thousand Islands (mid- and offshore): AB, Ayer Besar; UJ, Untung Jawa; R, Rambut; PS, Pari South; PN, Pari North; P, Panggang; C, Congkak; B, Bira.

The Kepulauan Seribu (Thousand Islands) stretch up to 80 km north of Jakarta and are comprised of 105 small (< 10 ha) and very low-lying (< 3 m above sea level) islands (Arifin, 2004). Indonesia’s first Marine National Park, the Thousand Islands National Park, was established in 1982 in the north of the island chain (Djohani, 1994). Most islands have lagoons and fringing reefs with reef development generally restricted to shallow depths (around 3–10 m, max. 20 m depth). The island chain is densely populated (total population: 22,700 people). A total of 65% of the people live on the four main islands Panggang, Pramuka, Kelapa and Harapan (Badan Pusat Statistik, 2012). Several rivers with a combined catchment area of 2,000 km^2^ discharge directly into JB and transport large amounts of untreated sewage and industrial effluents with high pollutant levels (Rees et al., 1999). The bay’s shoreline has been modified extensively over the last decades due to massive urbanization, industrialization and infrastructural development in Jakarta (60% of the shoreline) as well as due to agricultural or aquaculture developments (30% of the shoreline) (Bengen, Knight & Dutton, 2006). During the dry season, the predominantly south-easterly winds can cause polluted surface waters from the JB area to reach midshore reefs (definition see below), while during the wet season, north-westerly winds blow from offshore towards JB (Cleary, Suharsono & Hoeksema, 2006). In November 2012, during the transition time between northwest and southeast monsoon, eight coral reef sites across the Thousand Islands chain were visited. Three sites within JB (nearshore area; < 20 km) and five sites from the outer Thousand Islands (mid- and offshore area; 20–45 km and > 45 km, respectively) were chosen to represent both inhabited and non-inhabited islands. Reefs from the northern side or north-eastern side of each island (except for Pari South: here, the south side was included to account for the observed strong differences in coral cover between the northern and southern side of the island; (Abrar, Zamani & Wayan Nurjaya, 2011; Madduppa et al., 2012) were visited to ensure consistent wave exposure and current regimes (see Moll & Suharsono, 1986; Cleary, Suharsono & Hoeksema, 2006) (Table 1; Fig. 1).

**Table 1:** Description of sampling sites. Description of sampling sites (linear distance refers to distance from each site to the harbor Muara Angke in Jakarta).

Site	Site abbrev.	Longitude (E)	Latitude (S)	Linear distance to Jakarta (km)
Ayer Besar	AB	106°42.242	05°58.399	11.3
Untung Jawa	UJ	106°46.911	05°58.399	16.4
Rambut	R	106°41.597	05°58.202	17.3
Pari South	PS	106°36.963	05°52.094	31.4
Pari North	PN	106°37.440	05°51.001	32.6
Gosong Panggang	P	106°35.355	05°44.664	45.7
Gosong Conkak	C	106°35.274	05°42.303	49.5
Kayu Angin Bira	B	106°34.162	05°36.405	59.8

### Live benthic cover

Live benthic cover was determined at each site with 50 m line-intercept transects (n = 3) at 5 ± 0.5 m water depth (English, Wilkinson & Baker, 1994). Every two meters, on both sides of the transect line, high-resolution underwater photographs (n = 50 transect^−1^) were taken using a digital camera (Canon G12). A 1 × 1 m gridded quadrat frame was used for reference. These photographs were analyzed using CPCe software (Kohler & Gill, 2006) with 50 random points placed on each photo (Brown et al., 2004), and each point was assigned to one the following benthic categories: hard corals, *Nephthea* spp., *Sarcophyton* spp., other soft corals and macroalgae. Since corals are the principal structure-providing benthic organisms and the loss of this structure results in reduced diversity and functionality of the ecosystem (Stanley, 2003; Munday, 2004), the survey focused on corals. Overall total live coral cover was calculated as the sum of hard and soft coral cover. A detailed description of the survey as well as further data on other substrate types including sand, rubble and dead corals as well as macroalgae is given in Baum et al. (2015).

### Water quality

Anthropogenic stressors that reflect the water quality in the JB/Thousand Islands reef complex (De’ath & Fabricius, 2010; Fabricius et al., 2012) were determined at each sampling site. The water parameters temperature (°C), dissolved oxygen (DO; mg/L), pH, salinity (PSU), NTU and Chl *a* (μg/L) concentration of the water were measured at 1 and 3 m water depth, using a Eureka 2 Manta Multiprobe (Eureka Environmental Engineering, Austin, TX, USA). Water samples for inorganic nutrient analyses (nitrite (NO_2_), nitrate (NO_3_), phosphate (PO_4_), ammonia (NH_3_)) were taken at each sampling site at 1 and 4.5 m water depth. Dissolved inorganic nitrogen (DIN) is given as the sum of NO_2_, NO_3_ and NH_3_. Sedimentation rate was estimated by deploying sediment traps (as recommended by Storlazzi, Field & Bothner, 2011) at 5 ± 0.5 m depth for 22 ± 1 h at each site (n = 5 traps per site). For a detailed description of the sampling design and analysis of water parameters, refer to Baum et al. (2015).

### Photosynthetic yield and ETS activity of soft corals

At each site, fragments (∼5–10 cm length) of the two soft coral genera, *Sarcophyton* spp. and *Nephthea* spp. (recently synonymized with *Litophyton* by Van Ofwegen (2016), see http://www.marinespecies.org/aphia.php?p=taxdetails&id=205891), were sampled (n = 5) during SCUBA diving at ∼5 m water depth. These two soft coral genera were chosen due their high abundances along the island chain. At nearshore sites, a sufficient number in hard coral replicates was not available. Therefore, photosynthetic yield and ETS activity in hard corals could not be measured. Taxonomic identification in the field was performed based on Fabricius & Alderslade (2001) to genus, the lowest taxonomic level possible for field surveys. Fragments were always chosen with the same morphological appearance (e.g. the same color, type and length of tentacles, hardness, etc.) in order to minimize the collection of different species within the genera. *Sarcophyton* spp. samples were separated from the two morphologically similar-looking soft coral genera *Lobophytum* and *Sinularia* by considering that *Lobophytum* and *Sinularia* have “fingering” surfaces and that *Lobophyton* is harder than *Sarcophyton*.

#### Photosynthetic yield

Coral samples were placed immediately in two 100 L black plastic boxes, one box for each of the soft coral genera, respectively. The boxes were filled with fresh seawater from the sampling site. The water was aerated, and temperature, PSU, DO and pH monitored with a WTW 340i Multiparameter system (WTW, Germany) at regular intervals. 30% of the water was exchanged every 30 min. Corals were dark-adapted for 3 ± 24 h by covering the boxes with a lid (mean light in the box (PAR) = 4.3 PAR; measured with LI-COR Li400, Germany). Photosynthetic capacity was then determined by measuring the chlorophyll fluorescence of photosystem II (PS II), using a pulse-amplitude modulated fluorometer (DIVING-PAM, Walz, Germany). Photosynthetic yield (also called maximum quantum yield; F_v_/F_m_) (Walz, 1998) was measured by holding the sensor tip around 3–5 mm above the polyps (Rodolfo-Metalpa, Huot & Ferrier-Pagès, 2008). Number of fragments per site for both genera was n = 7, except for the sites Rambut (with n = 6 for *Nephthea* spp.), Ayer Besar (AB) and Bira (with n = 6 for *Sarcophyton* spp.), and Congkak (with n = 4 for *Sarcophyton* spp.).

#### Electron transport system (ETS) activity

Prior to dark-adaptation for measurement of photosynthetic yield, tissues samples were taken from each coral fragment, placed in small 2 ml glass vials and immediately stored in liquid nitrogen, until they could be placed in a −80 °C freezer. ETS activity was measured at ZMT in Bremen, Germany. Replicate number varied between the two genera: n = 5 for *Nephthea* spp. (except for the sites Untung Jawa (UJ), Rambut: n = 4 and Pari North, Bira: n = 3) and n = 4 for *Sarcophyton* spp. (except for the sites Pari North, Congkak, Bira: n = 3). The soft coral tissue samples (always kept on ice between steps) were ground with a plastic mortar for 90 s in homogenization buffer (HOM; stored at −20 °C) containing 1.5 mg/ml polyvinylpyrolidone (PVP), 75 μM MgSO_4_ × 7H_2_O and 0.2% Triton X-100 in 0.1 M phosphate buffer, pH 8.5 (following Owens & King, 1975). ETS enzyme extracts were prepared in a 50-fold volume (w:v) of homogenization buffer. After 1 min of tissue lysis by ultrasonication (Bandelin, Sonopuls HD 3100), the homogenates were centrifuged for 10 min at 2 °C and 1,500 g (Eppendorf, 5804 R). The resulting supernatant was transferred into a sterile Eppendorf cup and stored on ice until analyses. ETS activities were determined the same day, following Lannig et al. (2003) with slight modifications. The final assay volume was adjusted to 1 ml and the reaction mixture was prepared as follows in 1.5 ml single use plastic cuvettes: 500 μl assay buffer (0.1 M phosphate buffer, pH 8.5; stored at 4 °C) were mixed with 250 μl INT-solution (8 mM INT *(2-(4-Iodophenyl)-3-(4-nitrophenyl)-5-phenyl-2H-tetrazolium chloride)* in 0.1 M phosphate buffer, pH 8.5, stored at 4 °C) and 167 μl NADH-solution (7.2 mM NADH with 0.2% Triton X-100 (v:v) in 0.1 M phosphate buffer, pH 8.5, prepared daily), stirred with a plastic stirrer and incubated for 5 min at 30 °C in a cooling-thermomixer (HLC, MKR 23) in the dark. The reaction was started by adding 150 μl of sample homogenate to the assay mixture. Immediately afterwards, the increase in absorbance of ETS activity was measured at 490 nm for 5 min with a time interval of 15 s (applying the associated measuring software UV WinLab from Perkin Elmer) in a spectrophotometer (Perkin Elmer, Lambda 35). The resulting slope, calculated by subtracting the blank activity from sample activity, was further used to calculate enzymes activities. All samples were run in triplicate. ETS activity [*μmol O_2_ h^−1^ g^−1^*] was calculated according to the equation (Lannig et al., 2003):

(1) $$ETS-activity\;[\mu mol\;{O_2}\;{h^{-1}}\;{g^{-1}}] = {{\Delta A{{\min }^{-1}}} \over {\varepsilon \times d}} \times {{{V_{Assay}}} \over {{V_{Aliquot}}}} \times {{{V_{Extract}}} \over {{m_{sample}}}} \times R \times 60$$

ΔA min^−1^: change in sample absorbance–change in blank absorbance per min
ɛ: molar extinction coefficient of INTP Formazan [15,900 μl μmol^−1^ cm^−1^]
d: path length of the cuvette [1 cm]
*V_Assay_*: volume of the final assay mixture [1,000 μl]
*V_Aliquot_*: volume of homogenate used in the reaction mixture [150 μl]
*V_Extract_*: volume of the original homogenate [μl]
*m_sample_*: wet mass of the muscle tissue [g]
R: 0.5 (ratio of O_2_ to INT of 1:2)

### Statistical analysis

Differences among sites for any of the water quality parameters, benthic parameters or ETS activity rates, and photosynthetic yields for each of the two different soft coral genera, were analyzed using one-way ANOVA. In addition, differences between JB and outer Thousand Islands for hard and soft coral cover, respectively, were tested for using one-way ANOVA. All data were checked for assumptions of normality and homogeneity of variances. In case assumptions were not fulfilled, a Kruskal Wallis test was performed instead. If significant effects were detected, pairwise comparisons with the post-hoc Student-Newman-Keuls test were performed to assess significant differences between individual factors.

Linear regression analysis was performed to test whether gradual in- or decreases could be found in ETS activity and photosynthetic yield as well as benthic factors along the distance gradient from Jakarta. In addition, ETS activity and photosynthetic yield as well as benthic factors were checked for linear correlation with each other and with water factors, respectively. Linear regression with one breakpoint (i.e. two linear segments) was used instead when it was found to yield a higher correlation. Univariate statistics were performed with SigmaPlot 12.5.

Multivariate statistics were performed using PRIMER-E software v.6 (Clarke & Gorley, 2006). To account for different scales and units (Clarke & Ainsworth, 1993), the water factors PO_4_, NH_4_, NO_3_, NTU and Chl were log+1 transformed, followed by normalization of all water factors. All benthic factors were square root transformed (Clarke & Green, 1988). Bray-Curtis similarity matrices (Bray & Curtis, 1957) were calculated for the metabolic condition (ETS activity and photosynthetic yield) of *Sarcophyton* spp. and *Nephthea* spp., as well as the benthic cover and a Euclidian distance similarity matrix for water data (Clarke & Gorley, 2006). Distance-based redundancy analysis (dbRDA; Anderson, 2001) was used to visualize differences between sites. In addition, the role of individual stressors was assessed with the BEST routine (using the BioEnv procedure based on Spearman rank correlation; Clarke & Warwick, 2001) to determine which of the water and benthic factors best explained the metabolic condition and cover of *Sarcophyton* spp. and *Nephthea* spp.

## Results

### Live benthic cover

**Figure 2:**
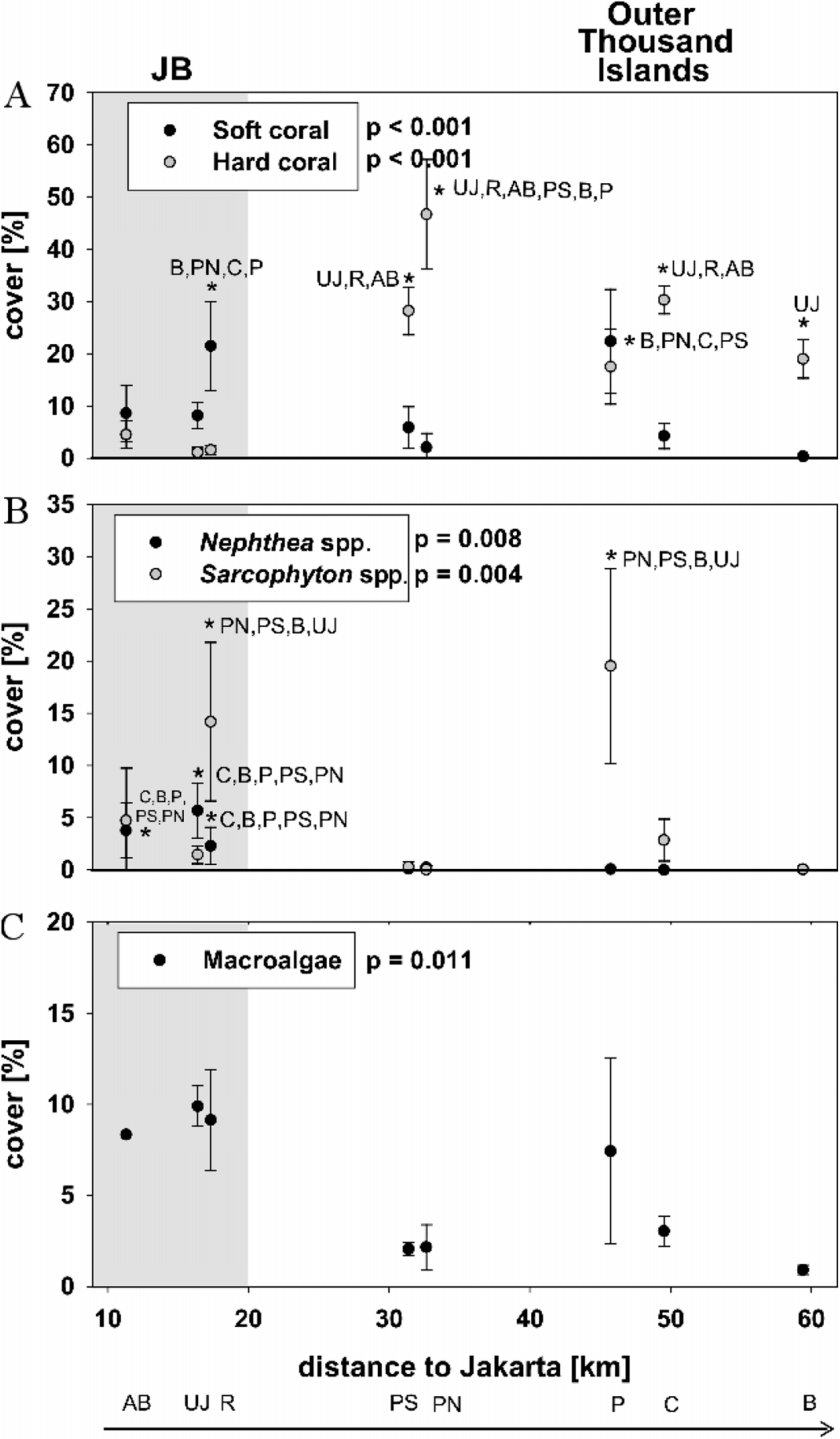
Live benthic cover. Mean cover (± SD) for hard and soft corals (A), the two soft coral genera *Sarcopyhton* spp. and *Nephthea* spp. (B) as well as macroalgae (C) for sites along the Thousand Islands (x-axis refers to distance to Jakarta). *p*-values (*p* > 0.05; one-way ANOVA) and significant post hoc results (*p* > 0.05; Student-Newman-Keuls) for differences among sites are given for each group. Consider different scales on y-axis. Study sites: AB, Ayer Besar; UJ, Untung Jawa; R, Rambut; PS, Pari South; PN, Pari North; P, Panggang; C, Congkak; B, Bira.

Hard coral cover was 2 ± 2% at nearshore sites and 28 ± 11% at the outer Thousand islands (mean ± SD). The highest hard coral cover (47 ± 11%) was found at Pari North in the midshore area. At nearshore sites mean soft coral cover (13 ± 6%) was significantly higher than hard coral cover (*p* = 0.023). Average soft coral cover at the outer Thousand islands was 7 ± 8%. Bruno et al. (2009) use a cut-off set at more than 50% cover of the dominant benthic taxa to define a phase shift. However, few reefs globally display such abundances (Hughes et al., 2010). Here we define “dominance” in terms of the category of corals (soft or hard) with the highest percent cover in relation to live benthic cover. Total coral cover was at all sites the largest group of live benthic cover (see Baum et al., 2015). Soft coral dominance occurred at all three nearshore sites. *Sarcophyton* spp. cover was significantly increased compared to *Nephthea* spp. cover at the two sites Rambut in JB and Panggang at the outer Thousand Islands (*p* < 0.05). Overall, soft coral cover along the Thousand Islands was highly patchy and mainly comprised of the genera *Nephtheidae* and *Xeniidae* as well as the family Alcyoniidae, of which nephtheids and alcyoniidids were dominating (see Table 2; Fig. 2).

**Table 2:** Benthic cover at each site. Mean cover (± SD) at each site (n = 3 transects per site) for hard and soft corals, the two soft coral genera *Sarcopyhton* spp. and *Nephthea* spp., macroalgae, other live as well as total coral cover for sites along the Thousand Islands.

Site		Hard coral (% cover)	Macroalgae (% cover)	Soft coral (% cover)	*Nephthea* spp. (% cover)	*Sarcophyton* spp. (% cover)	Total coral (% cover)	Other live (% cover)
**Jakarta Bay (JB)**	**AB**	5 ± 3	8 ± 2	9 ± 5	4 ± 2	5 ± 4	13 ± 2	4 ± 2
	**UJ**	1 ± 1	10 ± 1	8 ± 3	6 ± 2	2 ± 1	9 ± 3	3 ± 1
	**R**	2 ± 1	9 ± 3	22 ± 9	2 ± 2	14 ± 6	23 ± 8	2 ± 2
	**Mean**	2 ± 2	9 ± 2	13 ± 6	4 ± 2	7 ± 4	15 ± 4	3 ± 2
**Outer Thousand Islands**	**PS**	28 ± 5	2 ± 0	6 ± 4	0 ± 0	0 ± 0	34 ± 2	2 ± 1
	**PN**	47 ± 11	2 ± 1	2 ± 3	0 ± 0	0 ± 0	49 ± 9	1 ± 1
	**P**	18 ± 7	7 ± 5	22 ± 10	0 ± 0	20 ± 8	40 ± 11	2 ± 1
	**C**	30 ± 3	3 ± 1	4 ± 2	0 ± 0	3 ± 2	35 ± 2	6 ± 2
	**B**	19 ± 4	1 ± 0	0 ± 0	0 ± 0	0 ± 0	19 ± 4	7 ± 1
	**Mean**	28 ± 6	3 ± 1	7 ± 8	0 ± 0	5 ± 2	35 ± 6	4 ± 1

**Notes:**

Study sites: AB, Ayer Besar; UJ, Untung Jawa; R, Rambut; PS, Pari South; PN, Pari North; P, Panggang; C, Congkak; B, Bira.

Total soft coral cover did not show a significant linear trend with decreasing cover towards offshore, however the cover of *Nephthea* spp. significantly decreased towards north (*p* = 0.02). For *Sarcophyton* spp., no significant relation with distance to Jakarta could be found (Table 3).

**Table 3:** Correlations with environment (univariate analyses). Univariate analyses (linear regression) to test for correlations between the metabolic condition indicated by photosynthetic yield (F_v_/F_m_) and electron transport system (ETS) activity of the two soft coral genera *Sarcophyton* spp. and *Nephthea* spp. as well as the benthic cover with the distance to Jakarta, water factors and the cover of both soft coral genera. *p*-values are given.

Group			Correlation with (*p*-value)										
			Distance to Jakarta	Water parameters									Cover *Sarcophyton* spp./*Nephthea* spp.
				DIN	NH_3_	NO_2_	NO_3_	Sed	Chl *a*	Turb	PO_4_	Temp	
**Metabolic condition**	**Photosynthetic yield (F_v_/F_m_)**	*Nephthea* spp.	0.202	0.057	0.066	0.202	0.675	0.090	0.094	0.657	0.095	0.226	0.018
		*Sarcophyton* spp.	0.017	0.055	0.073	0.007	0.624	0.004*	0.267	0.267	0.180	0.898	0.849
	**ETS activity**	*Nephthea* spp.	0.846	0.023	0.017	0.376	0.455	0.255	0.629	0.934	0.009	0.038	0.379
		*Sarcophyton* spp.	0.681	0.107	0.09	0.441	0.346	0.087	0.464	0.982	0.057	0.143	0.274
**Benthic community (cover)**		*Nephthea* spp.	0.020	0.385	0.429	0.183	0.559	0.014	0.002	0.187	0.205	0.875	
		*Sarcophyton* spp.	0.894	0.117	0.107	0.381	0.607	0.854	0.956	0.315	0.516	0.643	
		Total soft coral	0.475	0.081	0.094	0.074	0.809	0.052	0.039	0.066	0.139	0.737	
		Total hard coral	0.060*	0.170	0.186	0.031	0.903	0.013	0.075	0.063	0.186	0.430	
		Macroalgae	0.190*	0.118	0.125	0.179	0.994	0.649	0.684	0.129	0.205	0.715	
		Total coral	0.030*	0.524	0.547	0.148	0.883	0.009	0.077	0.271	0.403	0.489	

**Note:**

* Refers to two linear segments (i.e. one breaking point).

Macroalgae cover was significantly different among sites and seemed higher at nearshore sites (mean 6 ± 5%) as well as at Panggang (mean 7 ± 5%) compared to sites from the outer Thousand Islands, however post hoc analysis did not show significant differences among sites. Neither did macroalgae cover show a significant decrease towards offshore (*p* = 0.19) (see Fig. 2; Table 2).

### Water quality

Most water parameters neither showed a clear separation of nearshore sites and sites from the outer Thousand Islands, nor a clear distance-based spatial pattern (i.e. with increasing distance to Jakarta), but rather localized patterns (see Baum et al. (2015) for further details). Water quality at nearshore sites in JB seemed generally worse than at sites from the outer Thousand Islands, with a 67% higher NTU (1.5 ± 0.7 NTU), 47% higher sedimentation rate (30.5 ± 0.4 g m^−2^ d^−1^), 44% higher DIN load (7.6 ± 3.6 μM/L) and Chl *a* (9.5 ± 4.5 μg/L) levels in the bay (mean ± SD); results were however not significant for all sites from JB. For other water parameters, e.g. the concentration of PO_4_ and NH_3_, values decreased towards offshore, with one exception. They showed significantly higher levels at one single offshore site (Panggang) compared to all other sites (*p* < 0.05) (see Table 4).

**Table 4:** Water quality. Data for sites in Jakarta Bay (JB) and outer Thousand Islands (see Baum et al. (2015) for details): Mean values (± SD) for the factors temperature (°C), pH, salinity (PSU), DO (mg/L), turbidity (NTU), sedimentation, the inorganic nutrients (µM/L) PO_4_, NO_3_, NO_2_, NH_4_ and Chl *a* (µg/L) at each site. The % difference between JB and outer Thousand Islands as well as *p*-values for differences between sites along the whole island chain (one-way ANOVA) and for linear regression analysis with distance to Jakarta are given for each factor.

Area		Tubidity (NTU)	Chl *a* (μg/L)	PO_4_ (μM/L)	NH_3_ (μM/L)	NO_2_ (μM/L)	NO_3_ (μM/L)	Sedimentation rate [g m^−2^ d^−1^]	DIN [μM/L]	Temperature (°C)	pH	Salinity (PSU)	DO
**Jakarta Bay (JB)**	**Mean**	1.49 ± 0.25	9.48 ± 1.27	2.36 ± 1.23	6.65 ± 1.32	0.42 ± 0.10	0.57 ± 1.16	30.39 ± 4.96	7.64 ± 0.87	30.47 ± 0.03	8.19 ± 0.01	32.41 ± 0.04	6.78 ± 0.23
	**AB**	0.73 ± 0.18	4.86 ± 0.57	4.09 ± 2.79	11.64 ± 2.36	0.5 ± 0.09	0.55 ± 0.10	31.02 ± 5.89	12.69 ± 0.40	30.48 ± 0.03	8.33 ± 0.00	32.24 ± 0.05	8.39 ± 0.17
	**UJ**	1.32 ± 0.26	15.77 ± 1.96	1.68 ± 0.73	4.62 ± 0.94	0.23 ± 0.11	0.67 ± 0.15	30.16 ± 4.72	5.52 ± 1.22	30.24 ± 0.05	8.09 ± 0.01	32.62 ± 0.04	5.54 ± 0.26
	**R**	2.4 ± 0.27	7.81 ± 1.30	1.31 ± 0.18	3.7 ± 0.65	0.53 ± 0.10	0.48 ± 0.24	30 ± 4.28	4.71 ± 1.00	30.68 ± 0.00	8.16 ± 0.00	32.36 ± 0.03	6.41 ± 0.26
**Outer Thousand Islands**	**Mean**	0.49 ± 0.20	1.76 ± 0.30	1.41 ± 0.35	3.64 ± 0.62	0.14 ± 0.04	0.48 ± 0.16	16.18 ± 4.96	4.25 ± 0.87	30.41 ± 0.02	8.15 ± 0.01	32.77 ± 0.04	6.64 ± 0.07
	**PS**	0.42 ± 0.27	1.48 ± 0.15	0.51 ± 0.08	2.82 ± 0.88	0.27 ± 0.04	1.02 ± 0.13	10.54 ± 2.60	4.11 ± 1.05	30.35 ± 0.03	8.14 ± 0.00	32.63 ± 0.03	6.59 ± 0.10
	**PN**	0.42 ± 0.14	2.84 ± 0.20	0.11 ± 0.02	0.46 ± 0.28	0.01 ± 0.01	0.65 ± 0.17	13.91 ± 3.18	1.11 ± 0.46	30.83 ± 0.00	8.18 ± 0.00	32.74 ± 0.03	6.50 ± 0.03
	**P**	0.54 ± 0.18	1.78 ± 0.41	4.35 ± 1.22	11.41 ± 0.73	0.16 ± 0.10	0.39 ± 0.10	14.41 ± 1.06	11.96 ± 0.94	30.14 ± 0.03	8.14 ± 0.00	32.95 ± 0.03	6.21 ± 0.07
	**C**	0.52 ± 0.24	0.89 ± 0.05	0.05 ± 0.05	2.03 ± 0.68	0.14 ± 0.02	0.16 ± 0.00	20.37 ± 3.50	2.33 ± 0.70	30.36 ± 0.03	8.18 ± 0.00	32.78 ± 0.03	6.58 ± 0.06
	**B**	0.54 ± 0.17	1.84 ± 0.70	2.04 ± 0.40	1.48 ± 0.54	0.1 ± 0.02	0.16 ± 0.18	21.65 ± 4.67	1.74 ± 0.76	30.34 ± 0.02	8.13 ± 0.00	32.74 ± 0.05	6.47 ± 0.10
**% difference JB and Outer Thousand Islands**		67	81.41	40.19	45.28	67.58	16.38	46.78	44.35	0.2	0.47	1.12	2.05
**One-Way ANOVA (*p*-value)**		0.005	0.003	< 0.001	< 0.001	< 0.001	< 0.001	< 0.001	< 0.001	0.114	0.083	0.007	0.106
**Correlation with distance to Jakarta (*p*-value)**		0.15	0.07	0.7	0.47	0.42	0.03	0.01	0.15	0.44	0.37	0.02	0.56

**Notes:**

Study sites: AB, Ayer Besar; UJ, Untung Jawa; R, Rambut; PS, Pari South; PN, Pari North; P, Panggang; C, Congkak; B, Bira.

### Photosynthetic yield

**Figure 3:**
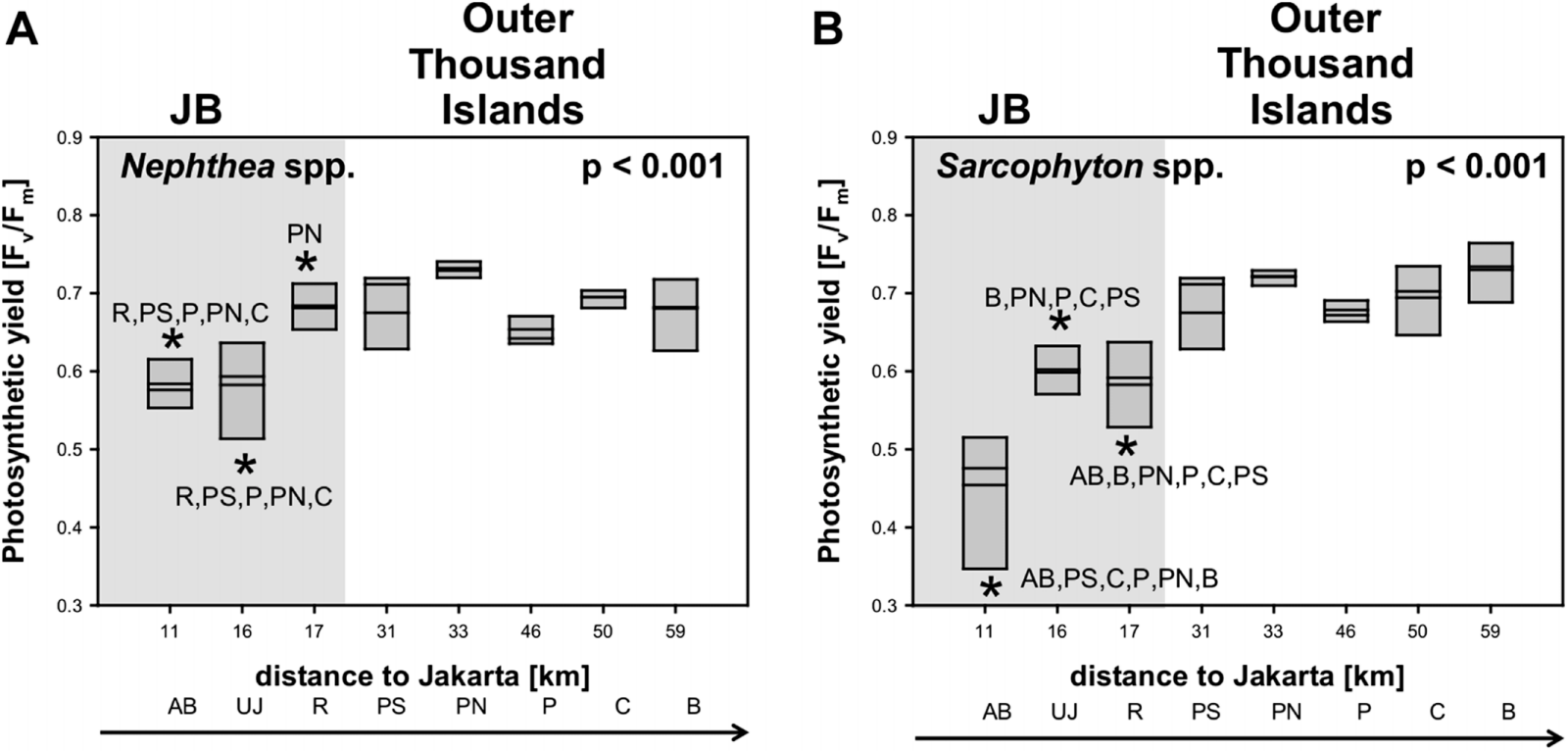
Mean electron transport system (ETS) activity *Nephthea* spp. and *Sarcophyton* spp. activity *Nephthea* spp. (A) and *Sarcophyton* spp. (B) for sites along the Thousand Islands (x-axis refers to distance to Jakarta). *p*-values (*p* > 0.05; one-way ANOVA) and significant post hoc results (*p* > 0.05; Student-Newman-Keuls) for differences among sites are given for each group. AB, Ayer Besar; UJ, Untung Jawa; R, Rambut; PS, Pari South; PN, Pari North; P, Panggang; C, Congkak; B, Bira.

Average photosynthetic yield (F_v_/F_m_) of *Sarcophyton* spp. (0.7 ± 0.1) and *Nephthea* spp. (0.7 ± 0.1) did not differ between the two genera. Significant differences in photosynthetic yield between sites were found for both soft coral genera (*p* < 0.001). Subsequent post hoc analysis revealed for *Sarcophyton* spp. that all sites in JB were significantly different from almost all other sites from the outer Thousand Islands (*p* < 0.05). Overall, the yield increased for *Sarcophyton* spp. towards the north (*p* = 0.017). Post hoc analysis for *Nephthea* spp. revealed a similar trend, with the two sites furthest south in the Bay (AB, UJ) being significantly different from most sites from the outer Thousand Islands (*p* < 0.05). However, the photosynthetic yield of *Nephthea* spp. did not significantly increase towards the north (*p* = 0.202) (Table 3; Fig. 3).

### ETS activity

**Figure 4:**
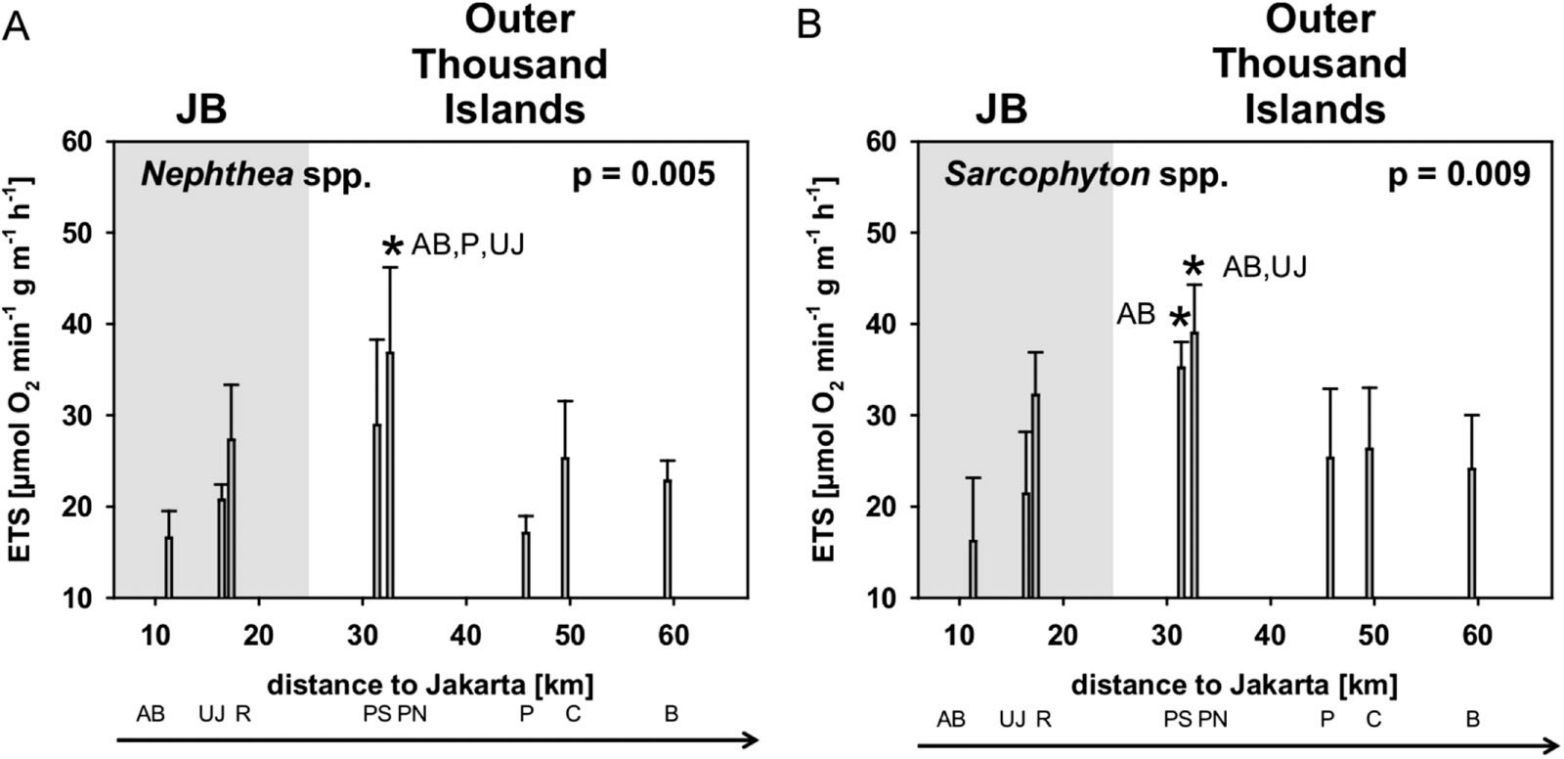
Mean electron transport system (ETS) activity *Nephthea* spp. (A) and *Sarcophyton* spp. (B) for sites along the Thousand Islands (x-axis refers to distance to Jakarta). *p*-values (*p* > 0.05; one-way ANOVA) and significant post hoc results (*p* > 0.05; Student-Newman-Keuls) for differences among sites are given for each group. AB, Ayer Besar; UJ, Untung Jawa; R, Rambut; PS, Pari South; PN, Pari North; P, Panggang; C, Congkak; B, Bira.

Average ETS-activity [*μmol O_2_ h^−1^ g^−1^*] of *Sarcophyton* spp. (25.8 ± 8.5) and *Nephthea* spp. (24.1 ± 6.8) did not differ between the two genera. Significant differences in ETS-activity among sites were found for *Nephthea* spp. (*p* = 0.005) and *Sarcophyton* spp. (*p* = 0.009). Subsequent post hoc analysis revealed for both genera that the two sites AB and UJ in JB were significantly different from the midshore site PN with the highest ETS-activity (Table 3; Fig. 4).

### Correlations between soft coral physiology and environment

The metabolic condition (indicated by photosynthetic yield and ETS) of both *Sarcophyton* spp. and *Nephthea* spp. was highly correlated with the overall water quality, with 79% of the variation in *Nephthea* spp. being explained by the three water parameters PO_4_, NH_3_ and temperature, and 68% of the variation in *Sarcophyton* spp. being explained by the three water parameters DO, pH and temperature. The correlation of the metabolic condition of both soft coral genera to live benthic cover was less significant, with 12% for *Nephthea* spp. and 6% for *Sarcophyton* spp. respectively. Along the Thousand Islands, 71% of overall live benthic cover could be linked to the water parameters NH_3_, NO_2_ and NTU. 39% of variation in the composite cover of both *Sarcophyton* spp. and *Nephthea* spp. could be explained by the differences in sedimentation rate and NH_3_ (see Table 5).

**Table 5:** Correlations with the environment (BioEnv test). Correlation between the metabolic condition indicated by photosynthetic yield (F_v_/F_m_) and electron transport system (ETS) activity of the two soft coral genera *Sarcophyton* spp. and *Nephthea* spp., respectively, and the water quality as well as live benthic cover. Data are based on the test BioEnv (correlation factors are shown).

Group		Correlation with			
		Water parameters		Live benthic cover	
		Corr	Factor	Corr	Factor
**Metabolic condition**	***Nephthea spp.***	0.79	PO_4_	0.12	*Sarcophyton* spp.
			NH_3_		Macroalgae
			Temp		Hard coral
	***Sarcophyton spp.***	0.68	DO	0.06	Macroalgae
			pH		*Nephthea* spp.
			Temp		Hard coral
**Benthic community**	**Overall**	0.71	NH_3_		
			NO_2_		
			Turb		
	**Cover of *Nephthea* spp. and *Sarcophyton* spp.**	0.39	Sed		
			NH_3_		

**Figure 5:**
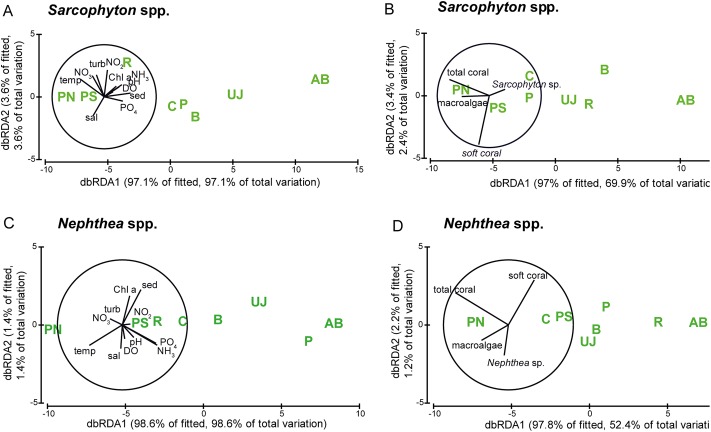
Visualization of the metabolic condition of *Sarcophyton* spp. and *Nephthea* spp. Visualization of the metabolic condition indicated by photosynthetic yield (F_v_/F_m_) and electron transport system (ETS) activity of the two soft coral genera *Sarcophyton* spp. and *Nephthea* spp. based on distance-based redundancy analysis (dbRDA). Water quality factors (A) *Sarcophyton* spp. and (C) *Nephthea* spp.) and benthic factors (B) *Sarcophyton* spp. and (D) *Nephthea* spp.) are overlain for both genera. Study sites: AB, Ayer Besar; UJ, Untung Jawa; R, Rambut; PS, Pari South; PN, Pari North; P, Panggang; C, Congkak; B, Bira.

The correlation of metabolic condition with water parameters as well as with benthic composition is visualized in Fig. 5 and shows a similar pattern for both genera. Sites however did not separate according to their distance to Jakarta, with the midshore site PN separated from the other sites and the nearshore site AB (see Fig. 5).

Photosynthetic yield of *Sarcophyton* spp. was significantly lower at sites with elevated sedimentation rates (*p* = 0.004) and NO_2_ (*p* = 0.007). ETS activity of *Nephthea* spp. was significantly lower at sites with elevated levels of DIN (*p* = 0.023), NH_3_ (*p* = 0.017) and PO_4_ (*p* = 0.009) as well as at higher temperatures (*p* = 0.038). The cover of *Nephthea* spp. was significantly higher at sites with higher Chl *a* (*p* = 0.0029) and sedimentation rate (*p* = 0.014).

Furthermore, at sites with a higher cover of *Nephthea* spp. a significantly higher photosynthetic yield of *Nephthea* spp. was measured (*p* = 0.018). Total soft coral cover was significantly higher at sites with higher Chl *a* concentrations (*p* = 0.039) (see Table 3).

## Discussion

Our findings suggest that water quality controls photosynthetic efficiency and ETS activity of dominant soft corals in JB, as well as the abundance of *Nephthea* spp. respectively. Findings revealed extremely eutrophic water conditions and overall dominance of soft corals within the bay compared to the outer Thousand Islands. Results indicate that both photosynthetic yield and ETS activity of the two common Indo-Pacific soft corals *Sarcophyton* spp. and *Nephthea* spp. were reduced in the bay and highly correlated with key water quality parameters, especially inorganic nutrient concentrations and sedimentation rates.

### Abundance of *Sarcophyton* spp. and *Nephthea* spp.

The reef condition along the Thousand Islands at shallow depths can be considered as being poor since total coral cover in most of the sites was < 25% (threshold based on Gomez & Yap, 1988). Especially in the bay, the loss in coral cover is highly dramatic, with a current cover below 5%. Currently, the highest hard coral cover can be found at midshore sites (47%), with a subsequent significant decrease towards offshore (mean cover: 17–30%) (data based on Baum et al., 2015). A similar pattern in hard coral cover along the distance gradient from the mainland was also observed by Cleary et al. (2014) for the Thousand Islands chain.

In this study, results indicate that soft coral dominance occurred at more sites within the bay than at the outer Thousand Islands. Within the bay, a mean cover of 2% hard and 13% soft corals was found compared to the outer Thousand Islands, where mean hard coral cover was 28% and that of soft corals was 7%. Overall, the cover of *Nephthea* spp. was significantly higher in JB compared to the outer Thousand Islands and decreased towards offshore, while the cover of *Sarcophyton* spp. generally was also higher within JB, but overall displayed a patchier distribution with very high abundances at the site Rambut in JB and at the offshore site Panggang.

### Water quality

Coral reefs along the Thousand Islands are exposed to numerous anthropogenic stressors that affect reefs both on regional and local scales (Zaneveld & Verstappen, 1952; DeVantier et al., 1998; Berkelmans et al., 2004; Selig et al., 2006; Burke et al., 2012). Findings from Baum et al. (2015) and this study reconfirm that the water quality is substantially decreased within the bay, with extremely eutrophic conditions compared to the outer Thousand Islands. In JB, PO_4_ levels reached 4 μM/L and DIN levels up to 13 μM/L. Other studies have reported similarly high values for eutrophication along the Thousand Islands, e.g. DIN levels of up to 21 μM/L (Ladwig et al., 2016) and total nitrogen of 54 μM/L as well as total phosphate levels of 5.2 μM/L (Van der Wulp et al., 2016). This extreme eutrophication may be the consequence of massive land runoff, lack of sewage treatment and large-scale agri- and aquaculture in the area. Along the Thousand Islands, overall Chl *a* levels (mean: 1.7 μg/L) were above the eutrophication threshold level of 0.2–0.3 μg/L (Bell, Lapointe & Elmetri, 2007) at all sites. Other significant stressors include increased sedimentation and NTU rates. Sites within JB on average had a 47% higher sedimentation rate compared to offshore sites in the Thousand Islands, with up to 30 g m^−2^ d^−1^. However, there is no clearly visible nearshore-offshore gradient in water quality. Along the outer Thousand Islands, water quality among sites is variable due to locally increased concentrations especially of inorganic nutrients at specific offshore sites, such as for example at Panggang, where PO_4_, NH_3_ and DIN concentrations peaked (see Baum et al., 2015).

Results from this study indicate that along the Thousand Islands, the live benthic cover composition was significantly related to anthropogenically-influenced water parameters. 71% of the variation in live benthic cover along the complete island chain could be linked to factors related to terrestrial run-off and eutrophication, especially NH_3_, NO_2_ and NTU. One of the main stress factors for coral reefs worldwide is eutrophication (Bell, Elmetri & Lapointe, 2014). Elevated concentrations of dissolved inorganic nutrients can reduce calcification rates in corals (Loya, 2004) and increase macroalgae cover (Stimson & Larned, 2000), thereby causing a decline in hard coral cover (see review by Fabricius, 2005).

### Physiology of *Sarcophyton* spp. and *Nephthea* spp.

Findings revealed that both photosynthesis and ETS activity of both soft coral genera were reduced in the bay. ETS activity and photosynthetic yield values measured in this study were comparable to those measured by other authors for different marine invertebrate species (Muscatine et al., 1984; Fanslow, Nalepa & Johengen, 2001; Ulstrup et al., 2011; Nahrgang et al., 2013).

For both *Sarcophyton* spp. and *Nephthea* spp., a relatively high correlation between their metabolic condition (indicated by photosynthesis and ETS activity) and the overall water quality was found. 79% of the variation in metabolic condition of *Nephthea* spp. was explained by PO_4_, NH_3_ and temperature, and 68% by DO, pH and temperature for *Sarcophyton* spp. Similarly, the cover of these two soft coral genera was linked to eutrophication-related stressors. The combined cover of both *Sarcophyton* spp. and *Nephthea* spp. along the whole island chain was explained to 40% by the water parameters sedimentation rate and NH_3_, with a generally higher cover at nearshore sites, especially of *Nepththea* spp.

To our knowledge, this is the first study measuring ETS activity in soft corals. We found reduced ETS levels in both genera at two nearshore sites characterized by high nutrient and sedimentation levels. The ETS activity of *Nephthea* spp. was significantly lower at increasing levels of DIN and significantly linked to changes in temperature. Several studies have proposed ETS activity as a useful complementary indicator of long-term metabolic activity, as it provides valuable information on the physiological status of organisms (Fanslow, Nalepa & Johengen, 2001; Nahrgang et al., 2013). Here, ETS activity was clearly linked to reduced water quality and indicates that ETS could be a useful stress indicator in soft corals.

Since both photosynthesis and ETS activity were highly negatively correlated with overall water quality, these results suggest a strong stress reaction towards the environmental conditions within the bay. Several other studies have reported decreased photosynthetic yields in corals affected by high levels of dissolved inorganic and particulate organic nutrients as well as NTU and sedimentation (e.g. Marubini, 1996). This could explain why photosynthetic yield and respiration, as indicated by ETS activity in this study, were lowest at the most eutrophic and turbid sites in this study. Metabolic condition of the two soft coral genera did however not increase linearly towards offshore, and thus did not reflect the distance to Jakarta and the improved water quality towards offshore. This may be due to a lack in a clear nearshore-offshore gradient in water quality as a result of locally increased concentrations of especially inorganic nutrients at specific offshore sites.

### Correlation between water quality and soft coral cover

Even though both *Sarcophyton* spp. and *Nephthea* spp. seem to be negatively affected by the reduced water quality in the bay, they occurred more frequently in the bay than hard corals. Heterotrophic filter-feeders such as many soft corals have been shown to benefit more from dissolved inorganic and particulate organic nutrients than hard corals (Fabricius & Dommisse, 2000; Fabricius, 2011). In areas of high particulate organic matter (POM), an important food source for soft corals (Fabricius & Dommisse, 2000), and elevated nutrient levels such as in JB, some soft corals can increase their heterotrophic feeding rates and thereby compensate for energy losses resulting from light reduction due to increased NTU. They may therefore be able to outcompete hard corals that thrive better in extremely low food and nutrient environments. Thus, soft coral dominance may be the result of release from competition with stony corals driven by water quality, particularly by eutrophication and sedimentation, and could be facilitated at nearshore sites in JB.

Nonetheless, in order to find out why both *Sarcophyton* spp. and *Nephthea* spp. are generally more abundant in JB, even though their metabolic conditions seem to be impaired, further investigations are required. It may be possible that hard corals in the area are more affected by the low water quality compared to soft corals. In order to assess whether soft corals are relatively better in tolerating the low water quality in the bay compared to hard corals, which could facilitate their dominance in the bay, comparable data on physiological responses of hard corals at the same study sites are needed. Further knowledge on the effects of declining water quality on the physiology of soft and hard corals such as growth rates, pigment concentrations as well as zooxanthellae densities are needed to determine whether the metabolism of soft corals is relatively more efficient under stressful conditions compared to hard corals. Long-term monitoring data is required to determine direct causal relationships between individual water stressors and stress responses. Overall, the metabolic response of soft corals is very complex, especially in areas with simultaneous exposure to different stressors such as along the Thousand Islands. The resulting final metabolic condition in soft corals under simultaneous exposure to many stressors, as was the case in this study, depends on the interactions of the various stressors. For example, it has been shown for hard corals that chronic exposure to dissolved inorganic nitrogen can reduce calcification rates and increase the concentrations of photopigments (Marubini & Davies, 1996) and photosynthesis rates (Fabricius, 2005). In contrast, shading due to high NTU and sedimentation rates of > 10 mg cm^−2^ d^−1^ (Rogers, 1990) have been shown to reduce photosynthesis in hard corals, which then may lead to reduced calcification (Anthony & Hoegh-Guldberg, 2003). Ban, Graham & Connolly (2014) provide a comprehensive review of multiple stressor interactions and found that in most studies investigating effects of several stressors, photosynthesis was reduced. Especially for the interpretation of ETS results in this study, it is necessary to know how ETS activity can change in response to individual water parameters and how similar organisms living in symbiosis with *Symbiodinium* spp. may react.

The results from this study also indicate a possible different ecology of *Sarcophyton* spp. and *Nephthea* spp., since each genera showed distinct patterns in its distribution. *Nephthea* spp. was significantly more abundant within JB and so may have been most opportunistic and able to benefit from conditions that were not optimal for it, but far more detrimental to other species, particularly stony corals. This genus may thus have had a higher tolerance towards the stressful conditions in JB compared to competing hard corals. *Sarcophyton* spp. though, while on average also being more abundant in JB, had two distinct local peaks. Under adverse conditions (JB), it was most abundant at the site with the lowest inorganic nutrients (particularly NH_3_ and DIN) within JB, Rambut. Thus, there may have been a threshold beyond which this genus could not cope with the overall bad water quality (i.e. beyond which it became heavily stressed). In contrast, in the Thousand Islands, where water overall was better, *Sarcophyton* spp. was most abundant at the site with the highest concentration of NH_3_ and DIN, Panggang. *Sarcophyton* spp. may have been generally less stressed in the Thousand Islands compared to JB, and thus may have benefitted from the locally nutrient-enriched waters at Panggang.

Ecological studies from the 1980s already predicted that shifts to soft-coral dominance could be expected after hard coral mass mortalities (e.g. after crown-of-thorns outbreaks) (Bradbury & Mundy, 1989). Even though alternative reef states with soft corals dominating the live benthic cover are not as common and widespread as coral-macroalgae phase shifts (e.g. Hughes, 1994), several studies have reported coral reefs in which the benthic community is dominated by soft corals locally in the Indo-Pacific (Robinson, 1971; Nishihira & Yamazoto, 1974; Endean, Cameron & DeVantier, 1988; Chou & Yamazato, 1990; Fabricius, 1998) and in the western Indian Ocean (Muhando & Mohammed, 2002). According to Fabricius (2011), shifts from hard to soft corals appear to be rare and restricted to productive, high-irradiance and wave-protected waters with strong currents, and zooxanthellate soft corals in particular are highly affected by NTU (Fabricius & De’ath, 2004). Neither the cover of *Sarcophyton* spp. nor of *Nephthea* spp. was however significantly affected by NTU rates within this study. Higher sedimentation rates and Chl *a* levels were positively related with higher abundances in the cover of *Nephthea* spp. Other studies found similar trends. For example, McClanahan & Obura (1997) observed that soft coral cover was higher at increased levels of sediment influence. Nonetheless, to deduce whether actually shifts to soft coral dominance have occurred in JB, long-term monitoring data is required. Cleary et al. (2008) found highly variable soft coral cover along the Thousand Islands in 1995, with a cover between 0% and 6% in the bay and up to 15% at some mid-and offshore sites. This indicates that soft coral cover may have increased in the bay, however further surveys over several years are necessary to confirm this.

Other confounding stressors that may have affected metabolic condition and shifts in benthic cover should be considered as well. Considering that sediments and water in JB have been reported to be contaminated with heavy metals (Rees et al., 1999; Williams, Rees & Setiapermana, 2000) and organic contaminants such as the insect repellent *N*,*N*-diethyl-*m*-toluamide (DEET) (Dsikowitzky et al., 2014), surfactants, pesticides and oil-related pollution (Rinawati et al., 2012; Baum et al., 2016), a possible toxic effect with inhibition of photosystem II and the mitochondrial electron transport chain could also explain the observed decreased rates in ETS activity and photosynthetic yield of soft corals in the bay compared to soft corals from reefs further north. A reduction in both ETS activity and photosynthetic yield rates after exposure to chemicals has been reported by several studies (e.g. Negri et al., 2005; Biscéré et al., 2015). Heavy metals can disturb the aerobic metabolism. For example, Maes et al. (2013) reported reduced ETS rates in fish after copper exposure. Similarly, herbicides and antifouling agents can cause a reduction in photosynthesis in corals (see review van Dam et al., 2011).

In addition, other factors such as the ability of both *Sarcophyton* spp. and *Nephthea* spp. to reproduce asexually, allowing them to spread over an area in which they are already present when competitors are removed (see Fabricius & Alderslade, 2001), as well as toxic and allelopathic features (Bakus, 1981; Coll et al., 1982; Tursch & Tursch, 1982; Sammarco et al., 1983; Maida, Sammarco & Coll, 1995; Fox et al., 2003) compared to hard corals may have additionally facilitated the observed soft coral dominance. For instance, *Nephthea* spp. produce natural products that have allelopathic capacities, and the production of two of these secondary metabolites has been linked to the eutrophication gradient along the Thousand Islands (Januar et al., 2011). Allelopathic features may also have affected abundances of *Sarcophyton* spp. At the offshore site Panggang, where relatively high nutrient concentrations and a significantly higher cover in *Sarcophyton* spp. was found compared to other sites from the outer Thousand Islands, the overall metabolic condition observed for *Sarcophyton* spp. was not significantly lower than in JB.

Another confounding factor influencing current distribution patterns of both soft coral genera may be impacts of the commonly practiced blast fishing along the outer Thousand Islands in the 1980s, which caused hard coral decline (Erdman, 1998). Fox et al. (2003) reported locally high abundances of the soft coral *Xenia* spp. (up to 80%) on coral rubble patches after chronic blast fishing practices in the Komodo National Park in eastern Indonesia. *Xenia* spp. are successful colonizers and have high fecundity and several dispersal modes (Benayahu & Loya, 1985). Further studies should assess how both *Sarcophyton* spp. and *Nephthea* spp. are affected by the aftermath of blast fishing practices. Currently, it is not fully understood in what way shifts to soft coral dominance may be triggered by pulse disturbances (e.g. blast fishing) as top-down control and whether a loss of resilience caused by factors not considered here preceded this proximal trigger (see review by Norström et al., 2009). Further studies on how top-down control may act as a driver on soft coral dominance along the Thousand Islands are needed.

## Conclusions and Outlook

Results in this study suggest that water quality, particularly eutrophication, could cause soft coral dominance in JB. Water quality has to be improved in order to prevent or reverse further phase shifts in the area. Even though this study is not able to determine direct causal relationships between individual stressors and changes in the ETS activity and photosynthetic yield of both *Nephthea* spp. and *Sarcophyton* spp., the current study indicates that the metabolic condition of both soft coral genera is affected by reduced water quality (and other anthropogenic stressors), and that ETS activity and photosynthetic yield may be useful indicators of overall metabolic condition and stress level. Future investigations should measure the responses of individual species within the two soft coral genera used in this study to test whether these species show similar physiological responses. While every effort was made to sample specimens of the same external appearance in each of the genera, in some cases, specimens of a similar-looking but different species of the same genus may have been sampled. Therefore, physiological results from this study need to be reconfirmed. Currently, there is still a lack in knowledge on physiological processes and compensating mechanisms of soft corals exposed to environmental stressors, however such knowledge is essential if the processes involved in shifts of benthic reef communities dominated by hard corals to those dominated by soft corals is to be understood. Data on respiration and photosynthesis should be combined with data on energy reserves (lipids, proteins etc.) in both hard and soft corals in order to determine cellular energy allocation during stress (Novais & Amorim, 2013). In addition, parallel to metabolic measurements, other ecological factors, such as reproductive capacity of the involved soft corals, as well as growth rates and pigment concentrations, should be determined to understand mechanisms involved in phase shifts. Management of coral reefs requires an understanding of the conditions under which phase shifts to different states occur. When considering the importance of coral reefs for the livelihoods of millions of people in developing countries, the need for more effective coral reef management is obvious.

## Supplemental Information

10.7717/peerj.2625/supp-1 Supplemental Information 1 Raw data (sedimentation rates, nutrient concentrations, other water parameters, benthic cover, ETS activitiies and photosynthetic yields) applied for data analyses and preparation for Figs. 2–5 and Tables 2–5.

10.7717/peerj.2625/supp-2 Supplemental Information 2 Comparison of electron transport system (ETS) activity, photosynthetic yield (F_v_/F_m_) and benthic cover between sites. Table S1. Comparison of electron transport system (ETS) activity, photosynthetic yield (F_v_/F_m_) and benthic cover between sites (One-Way Anova and post hoc Student Newman-Keuls Method). Study sites: AB, Ayer Besar; UJ, Untung Jawa; R, Rambut; PS, Pari South; PN, Pari North; P, Panggang; C, Congkak; B, Bira. Replicate number varied between the two species for the ETS-activity: n = 5 for *Nephthea* spp. (except for the sites UJ, R: n = 4 and PN, B: n = 3) and n = 4 for *Sarcophyton* spp. (except for the sites PN, C, B: n = 3). For photosynthetic yield n = 7 per fragment was used (except for the sites R (*Sarcophyton* spp. and *Nephthea* spp.) with n = 6 and UJ (*Sarcophyton* spp.) with n = 4).

## Additional Information and Declarations

### Field Study Permissions

The following information was supplied relating to field study approvals (i.e., approving body and any reference numbers): A research permit was granted by The State Ministry of Research and Technology, Republic of Indonesia (permit number: 2C11JD5292-L).

### Data Deposition

The following information was supplied regarding data availability: The raw data has been supplied as Supplemental Dataset Files.
