# Supplementary material for: Abundance and physiology of dominant soft corals linked to water quality in Jakarta Bay, Indonesia
Source: PeerJ. 2016 Nov 29;4:e2625. doi: 10.7717/peerj.2625 (PMC5127238; doi:10.7717/peerj.2625)
Supplement: Supplemental Information 2 — Table S1. Comparison of electron transport system (ETS) activity, photosynthetic yield (Fv/Fm) and benthic cover between sites (One-Way Anova and post hoc Student Newman-Keuls Method). Study sites: AB, Ayer Besar; UJ, Untung Jawa; R, Rambut; PS, Pari South; PN, Pari North; P, Panggang; C, Congkak; B, Bira. Replicate number varied between the two species for the ETS-activity: n = 5 for Nephthea spp. (except for the sites UJ, R: n = 4 and PN, B: n = 3) and n = 4 for Sarcophyton spp. (except for the sites PN, C, B: n = 3). For photosynthetic yield n = 7 per fragment was used (except for the sites R (Sarcophyton spp. and Nephthea spp.) with n = 6 and UJ (Sarcophyton spp.) with n = 4). [file peerj-04-2625-s002.docx]

| **Factor** | **Genera** | **Test** | **DF** | **SS** | **MS** | **F** | ***p*-value** | **Post-hoc (Student-Newman-Keuls Method)** |
| --- | --- | --- | --- | --- | --- | --- | --- | --- |
| **ETS** | *Nephthea* spp. | One-Way-ANOVA | 7 | 1209.8 | 172.8 | 3.97 | 0.005 | PN vs. AB,P, UJ |
|  | *Sarcophyton* spp. | One-Way-ANOVA | 7 | 1309.4 | 187.1 | 3.71 | 0.009 | PN vs. AB,UJ,AB |
|  |  |  |  |  |  |  |  | PS vs. AB |
| **Photosynthetic yield** | *Sarcophyton* spp. | One-Way-ANOVA | 7 | 142577.3 | 20368.2 | 10.88 | <0.001 | R vs. AB |
|  |  |  |  |  |  |  |  | B vs. AB,R,UJ |
|  |  |  |  |  |  |  |  | PN vs. AB,R,UJ |
|  |  |  |  |  |  |  |  | P vs. AB,R,UJ |
|  |  |  |  |  |  |  |  | C vs. AB,R,UJ |
|  |  |  |  |  |  |  |  | PS vs. AB,R,UJ |
|  |  |  |  |  |  |  |  | UJ vs. AB |
|  | *Nephthea* spp. | Kruskal-Wallis Test |  |  |  |  | <0.001 | R vs. UJ,AB |
|  |  |  |  |  |  |  |  | PS vs. UJ,AB |
|  |  |  |  |  |  |  |  | P vs. UJ,AB |
|  |  |  |  |  |  |  |  | PN vs. UJ,AB,R |
|  |  |  |  |  |  |  |  | C vs. UJ,AB |
| **Benthic cover** | *Sarcophyton* spp. | Kruskal-Wallis Test |  |  |  |  | 0.004 | P vs. PN,PS,B,UJ |
|  |  |  |  |  |  |  |  | R vs. PN, PS,B,UJ |
|  | *Nephthea* spp. | Kruskal-Wallis Test |  |  |  |  | 0.008 | UJ vs. C,B,P,PS,PN |
|  |  |  |  |  |  |  |  | AB vs. C,B,P,PS,PN |
|  |  |  |  |  |  |  |  | R vs. C,B,P,PS,PN |
|  | Total soft coral | One-Way-ANOVA | 7 | 1472 | 210.3 | 7.16 | <0.001 | P vs. B,PN,C,PS,UJ,AB |
|  |  |  |  |  |  |  |  | R vs. B,PN,C,PS,UJ,AB |
|  | total hard coral | One-Way-ANOVA |  |  |  |  | <0.001 |  |
|  | Macroalage | One-Way-ANOVA |  |  |  |  | 0.011 | - |
